# Single shot three‐dimensional pulse sequence for hyperpolarized ^13^C MRI

**DOI:** 10.1002/mrm.26168

**Published:** 2016-02-24

**Authors:** Jiazheng Wang, Alan J. Wright, De‐en Hu, Richard Hesketh, Kevin M. Brindle

**Affiliations:** ^1^ Cancer Research UK Cambridge InstituteUniversity of Cambridge, Li Ka Shing Centre, Robinson WayCambridgeUnited Kingdom; ^2^ Department of BiochemistryUniversity of CambridgeTennis Court RoadCambridgeUnited Kingdom.

**Keywords:** metabolism, imaging, tumors, pyruvate, lactate, spiral trajectory

## Abstract

**Purpose:**

Metabolic imaging with hyperpolarized ^13^C‐labeled cell substrates is a promising technique for imaging tissue metabolism in vivo. However, the transient nature of the hyperpolarization, and its depletion following excitation, limits the imaging time and the number of excitation pulses that can be used. We describe here a single‐shot three‐dimensional (3D) imaging sequence and demonstrate its capability to generate ^13^C MR images in tumor‐bearing mice injected with hyperpolarized [1‐^13^C]pyruvate.

**Methods:**

The pulse sequence acquires a stack‐of‐spirals at two spin echoes after a single excitation pulse and encodes the kz‐dimension in an interleaved manner to enhance robustness to B_0_ inhomogeneity. Spectral‐spatial pulses are used to acquire dynamic 3D images from selected hyperpolarized ^13^C‐labeled metabolites.

**Results:**

A nominal spatial/temporal resolution of 1.25 × 1.25 × 2.5 mm^3^ × 2 s was achieved in tumor images of hyperpolarized [1‐^13^C]pyruvate and [1‐^13^C]lactate acquired in vivo. Higher resolution in the z‐direction, with a different k‐space trajectory, was demonstrated in measurements on a thermally polarized [1‐^13^C]lactate phantom.

**Conclusion:**

The pulse sequence is capable of imaging hyperpolarized ^13^C‐labeled substrates at relatively high spatial and temporal resolutions and is robust to moderate system imperfections. Magn Reson Med 77:740–752, 2017. © 2016 The Authors Magnetic Resonance in Medicine published by Wiley Periodicals, Inc. on behalf of International Society for Magnetic Resonance in Medicine. This is an open access article under the terms of the Creative Commons Attribution License, which permits use, distribution and reproduction in any medium, provided the original work is properly cited.

## INTRODUCTION

The enormous gain in spin polarization afforded by dissolution dynamic nuclear polarization has enabled dynamic measurements of tissue metabolism in vivo using ^13^C magnetic resonance spectroscopic imaging following intravenous injection of hyperpolarized ^13^C‐labeled substrates 1, 2, 3, 4. Measurements of the labeled substrates and their products can be used to measure various metabolic fluxes and enzymatic processes. However, this is demanding in terms of imaging speed due to the short lifetime of the hyperpolarization 5. For example, hyperpolarized [1‐^13^C]pyruvate has a T_1_ in vivo of approximately 40 s 6, which means that the signal is observable for only approximately 3 min. The imaging technique must be sufficiently fast to sample the dynamic changes in metabolite labeling within this narrow time window 7.

Various techniques have been proposed to increase imaging speed. Echo planar spectroscopic imaging (EPSI), with a dual‐spin‐echo scheme, can produce a two‐dimensional (2D) spectroscopic image and an entire spectrum at each pixel, with a temporal resolution of several seconds 8, 9, 10, 11. The EPSI technique can be extended to cover 3D‐spatial‐1D‐spectral k‐space and has been further accelerated by incorporating compressed sensing 12, 13, 14. Spectroscopic imaging methods have also been proposed that exploit spiral trajectories 15, 16, 17, which achieve higher imaging efficiency, although at the cost of increased vulnerability to system imperfections. Fast imaging can also be facilitated by the inclusion of spectral‐spatial (SpSp) excitation pulses. Because the spectra obtained following injection of a hyperpolarized substrate are relatively sparse, it is not necessary to acquire a full spectrum. For example, in many tumor‐bearing animals injected with hyperpolarized [1‐^13^C]pyruvate the tumor spectra are dominated by resonances from pyruvate and lactate 6.

SpSp pulses can be used to excite alternately the target metabolites, followed by an echo planar readout to acquire separate images from each metabolite 18, 19, 20, 21. These pulses have the advantage that different flip angles can be used for each metabolite, for example in the case of pyruvate, where the signal is intense, a low flip angle pulse can be used to preserve the polarization, while in the case of lactate, where the signal may be much smaller, a larger flip angle can be used to increase the signal‐to‐noise ratio (SNR) 3. A disadvantage of the technique is that imperfections in the chemical shift selectivity of the pulse can lead to contamination of the images from the less abundant metabolites with signal from the more abundant metabolites 18. Another approach to fast imaging is based on the Dixon method 22, which exploits the sparsity in the spectral dimension by acquiring a small number of 2D images rather than a full free induction decay at each spatial point.

Heterogeneity is an intrinsic property of tumors 23, and can be correlated directly with the effectiveness of treatment 24. 3D imaging is desirable, therefore, both to investigate tumor heterogeneity and to monitor fully the response of a tumor to treatment. An inherent limitation of the imaging techniques described above is that, for full 3D coverage, they require several excitations for each image, which will deplete the polarization and thus accelerate signal decay 5. This is the case for 3D imaging techniques, where an extra excitation is required for each phase encoding step in the slice direction and is also true for the 2D imaging techniques, where slice cross‐talk, due to an imperfect excitation profile, can accelerate polarization decay.

We have developed a novel single‐shot 3D imaging sequence with SpSp excitation and a hybrid dual‐spin‐echo‐spiral readout. The sequence was designed to minimize loss of polarization by reducing the number of excitation pulses required, while maintaining high spatial and temporal resolutions. The sequence was demonstrated on both phantoms and mice and its robustness to B_0_ field inhomogeneity, a common challenge for fast imaging techniques, was also demonstrated theoretically.

## METHODS

### Tumor Model

Animal experiments were performed in compliance with a project license issued under the Animals (Scientific Procedures) Act of 1986. Protocols were approved by the Cancer Research UK, Cambridge Institute Animal Welfare and Ethical Review Body. Female C57BL/6J mice were injected subcutaneously in the lower flank with 5 × 10^5^ EL4 lymphoma cells. The tumors were allowed to grow for 8–11 days, when they achieved a size of 1–1.7 cm in diameter. The mice were fasted for 6–8 h before imaging.

### Hyperpolarized [1‐^13^C]pyruvate

The sample contained 44 mg [1‐^13^C]pyruvic acid (CIL, MA), 15 mM OXØ63 (GE Healthcare, Amersham, UK), and 1.4 mM gadoterate meglumine (Dotarem; Guerbet, Roissy, France) and was hyperpolarized using a Hypersense Polarizer (Oxford Instruments, Abingdon, UK) at 1.2 K in a magnetic field of 3.35 Tesla (T), with microwave irradiation at 94.126 GHz. The sample was then rapidly dissolved in 6‐mL buffer containing 40 mM Tris, 185 mM NaOH, and 100 mg/L EDTA heated to 180 °C and pressurized to 10 bar.

### MRI Scanner

Experiments were performed at 7T (Agilent, Palo Alto, CA). The maximum gradient strength was 40 Gauss/cm and slew rate 3000 T/m/s. A 42‐mm‐diameter bird‐cage volume coil for ^1^H transmission and reception and a similar volume coil for ^13^C transmission and a 20‐mm‐diameter surface coil for ^13^C detection were used (Rapid Biomedical GMBH, Rimpar, Germany).

### Spectral‐Spatial Pulse

**Figure 1:**
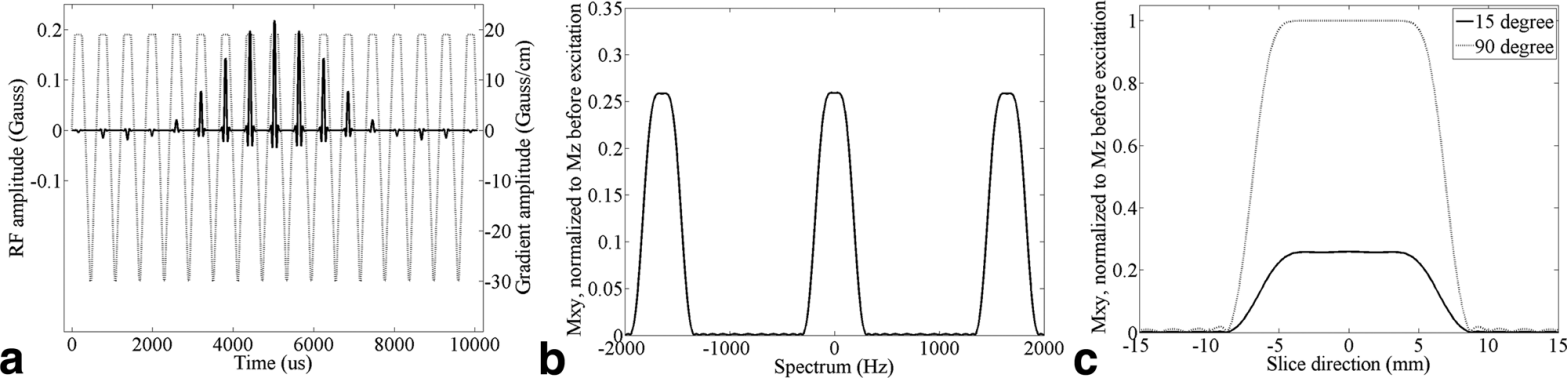
Spectral‐spatial excitation pulse to excite alternately the [1‐^13^C]pyruvate and [1‐^13^C]lactate resonances. **a**: RF profile (in Gauss) and oscillating slice selection gradient (in Gauss/cm). **b**: Frequency response (15 ° at 0 Hz). **c**: Slice profiles for 15 ° and 90 ° flip angles. Note that although the design target of the pulse was 15 °, the quality of the excitation profile was maintained for the 90 ° flip angle pulse.

The SpSp pulse (Fig. 1a) was designed to detect [1‐^13^C]pyruvate and [1‐^13^C]lactate, without disturbing the polarization of alanine and pyruvate‐hydrate. The pulse had a bandwidth at half maximum of 350 Hz and a distance between the excitation bands of 1645 Hz (Fig. 1b). The pulse design followed the small tip‐angle approximation 25 to give a flip angle of 15 ° with a peak B_1_ of 0.2185 Gauss. The flip angle could be increased to 90 ° when a peak B_1_ of 1.3111 Gauss was used (Fig. 1c). The radofrequency (RF) profiles in the spectral and the spatial dimensions were designed using the SLR algorithm 26. The pulse duration was 10.056 ms and the sampling time for both RF and gradient waveforms was 4 μs. Because the pulse was designed for 3D imaging, it relaxed the design demands on minimum slice thickness, which was set to 1.2 cm to leave more flexibility in other parameters, such as sub pulse excitation profiles and spectral stopband width. Simulations showed a full width at half maximum (FWHM) for the RF response of 1.3 cm. A fly‐back design enhanced the pulse's robustness to system imperfections, where only the positive gradient lobes, at maximally 19 Gauss/cm, were accompanied by the RF sub pulses. The negative gradient lobes were designed to have the smallest duration, with a maximal gradient strength of ‐29.925 Gauss/cm. The sub pulse had a time‐bandwidth product of 6. For the spectrally selective pulse, this was 3.52. The RF pulse and gradient waveforms were generated using custom‐written Matlab (The Math Works, Natick, MA) scripts.

### Pulse Sequence

**Figure 2:**
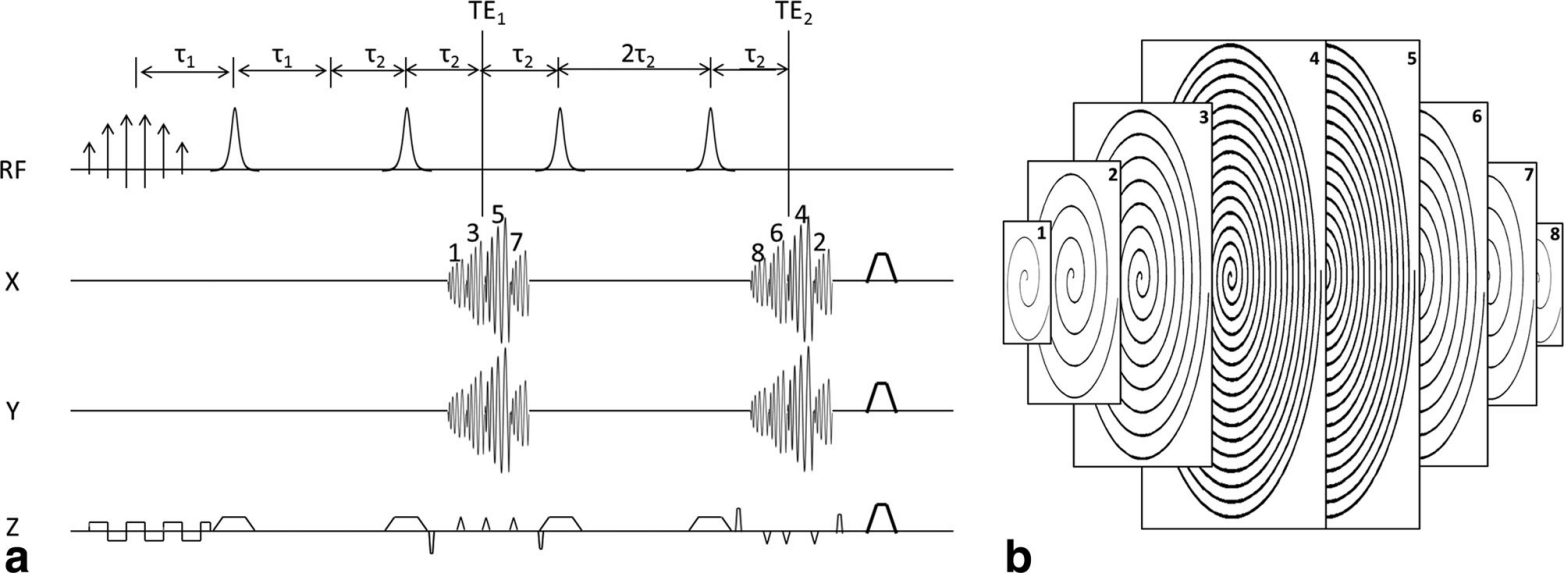
The pulse sequence (**a**) and its 3D k‐space trajectory (**b**). The pulse sequence includes a spectral‐spatial excitation pulse and two pairs of adiabatic inversion pulses. All phase encoding steps in the z‐direction were completed after a single excitation by using blipped gradients. Two spin echoes were acquired in two groups of spiral readouts, resulting in dual‐T_2_ weighted contrast. The gradients within each acquisition interval were self‐refocused, and the sequence ends with spoiler gradients along all three axes. The whole of k‐space was acquired as a stack of interleaved spirals. The 1^st^, 3^rd^, 5^th^, and 7^th^ spirals were acquired in the first group, while the 8^th^, 6^th^, 4^th^, and 2^nd^ spirals were acquired in the second group. The kx‐ky matrix size is larger in the center of the kz‐direction and smaller in the peripheral planes, resulting in a spherical 3D k‐space.

The pulse sequence (Fig. 2a) acquires a spherical stack of spirals in 3D k‐space (Fig. 2b), such that each kx‐ky plane has a spiral‐out trajectory. Two groups of four spirals are acquired, where the four spirals in the first group acquire data for the 1^st^, 3^rd^, 5^th^, and 7^th^ positions in the kz‐direction, while the second group acquires the 8^th^, 6^th^, 4^th^, and 2^nd^ positions. For spirals 1 to 8, the corresponding Cartesian matrix sizes were 4 × 4, 8 × 8, 16 × 16, 32 × 32, 32 × 32, 16 × 16, 8 × 8, and 4 × 4, respectively. The duration of the 4 × 4, 8 × 8, 16 × 16, and 32 × 32 spirals (including the rewinding gradients) were 0.656, 1.344, 3.240, and 8.580 ms, respectively, and were designed to achieve the same field of view (FOV) 27. To encode spatial information in the slice direction, each blip between spirals traverses two steps in the kz‐direction. There are pre‐ and re‐phasing gradients so that the z‐axis gradient, in each acquisition interval, is self‐refocused. Because each spiral is also self‐refocused on the x and y axes, the encoding gradients on all three axes are fully balanced in each acquisition interval.

Imperfect refocusing pulses, due to B_0_ and B_1_ field inhomogeneity, can deplete the polarization very quickly. Therefore, adiabatic pulses with a hyperbolic‐secant (HS) profile were used. Because a single HS adiabatic inversion pulse would leave a quadratic phase across the swept frequency band, this requires the HS pulse to be played out in pairs, hence the sequence shown in Figure 2a. For the spiral trajectory in Figure 2b, τ_1_ was 10 ms and τ_2_ was 15 ms. The two central spirals (4^th^ and 5^th^) were acquired at the spin‐echo time so that the center of k‐space was free from the effects of B_0_ field inhomogeneity. This resulted in two TE times, where TE_1_ was 50.6 ms and TE_2_ was 110.504 ms. Spoiling gradients on all three axes at the end of the sequence de‐phase residual transverse magnetization.

The pulse sequence can be adapted to other k‐space trajectories, depending on the required resolutions in the x–y plane and z‐direction. Two alternative designs were tested. A 16 × 16 × 8 design (compared with the original 32 × 32 × 8 design) consisted of eight spirals (again two sets of four) where each had a 16 × 16 matrix in the x–y plane. In the second design (16 × 16 × 12), 12 spirals were divided into two groups to achieve 12 phase encoding steps in the z‐direction, while each spiral encoded a 16 × 16 matrix in the x–y plane. In these alternative designs, the overall 3D k‐spaces are cylindrical, in contrast to the more‐spherical k‐space in the original sequence.

Theoretical point spread functions (PSFs), ignoring relaxation, were simulated as described by Durst et al 28. A constant k‐space was sampled by each of the proposed acquisition schemes and a 3D Fourier transform was performed to yield the PSFs. The sampled k‐spaces were zero‐filled (32 × 32 × 8 to 512 × 512 × 128, 16 × 16 × 8 to 512 × 512 × 256, 16 × 16 × 12 to 512 × 512 × 384) before Fourier transformation.

### Phantom Imaging

All three sequences were tested on a cylindrical phantom (7 mm inner‐diameter) filled with thermally polarized 8.5M [1‐^13^C]lactate. For each design, a series of FOVs in the z‐direction (16 mm, 20 mm, 24 mm, 32 mm, and 40 mm) were acquired to determine the slab profile. The nominal in‐plane resolutions were 1.25 mm, 2.5 mm, and 2.5 mm for the 32 × 32 × 8, 16 × 16 × 8, and 16 × 16 × 12 designs, respectively. The nominal slice thickness could be calculated as the z‐direction FOV divided by the number of phase encoding steps. The SpSp pulse was increased to a flip angle of 90 ° to excite a slab of 13 mm in all phantom experiments. Each acquisition was accompanied by a reference image in which the encoding gradients on the z axis were turned off.

T_2_ weighted proton images were acquired with a fast spin echo (FSE) sequence, to provide a positional frame of reference, with a FOV of 40 mm and matrix size 256 × 256, with eight slices covering 20 mm in the z‐direction.

### Dynamic Imaging In Vivo

Pyruvate and lactate images were acquired in alternating order and 30 pairs of images were acquired in total (60 s total acquisition time). The excitation pulse was set alternately at the pyruvate and lactate resonance frequencies. Each pair of acquisitions were 1 s apart, to give a “frame rate” of one every 2 s per metabolite. The z‐axis blipping and their prephasing and rewinding gradients, were turned off for the 8^th^ pair of acquisitions and the data used as a phase‐reference. Hyperpolarized [1‐^13^C]pyruvate was injected after the first image was acquired. A flip angle of 15 ° was used for pyruvate and 90 ° for lactate so that the lactate acquisition followed a saturation‐recovery scheme 20. The FOV in the x–y plane was 40 mm, with a matrix size of 32 × 32. The FOV in the z‐direction was 20 mm, resolved into eight pixels. The nominal spatial resolution was, therefore, 1.25 × 1.25 × 2.5 mm^3^. The nominal excitation slab thickness was 13 mm, smaller than the z‐direction FOV to avoid image wrap‐around in the z‐direction. The frequencies of the selective excitation pulses were calculated from the measured water proton frequency, based on prior measurements of the [1‐^13^C]lactate, [1‐^13^C]pyruvate and water proton resonance frequencies. The [1‐^13^C]pyruvate resonance frequency was at ‐916 Hz from the [1‐^13^C]lactate resonance frequency.

T_2_ weighted proton images were acquired for positional reference. These were 40 × 40 mm^2^ (256 × 256) FSE images with a slice thickness of 2.5 mm. The 2D spoiled gradient echo proton images with varying TEs (1.6, 1.7, 1.8, and 1.9 ms, respectively) were also acquired with the same FOV and slice thickness, and a matrix size of 32 × 32. A 3D phase unwrap was applied across the xy‐plane and the slice axis and the phase maps converted to a ΔB_0_ map based on the differences in TE. These were used to estimate the B_0_ field homogeneity in the imaging volume of the hyperpolarized ^13^C experiment.

### Image Reconstruction

A phase correction was performed, similar to that described in Lai and Glover 29. Interspiral phase variations were measured by comparing the leading data points between spirals in the reference scan. The measured phase variations were then subtracted from the regular imaging spirals. Corrected data for each spiral were gridded onto a Cartesian plane 30, and a 3D Fourier transform was applied. Phase correction and image reconstruction were performed in Matlab.

## RESULTS

**Figure 3:**
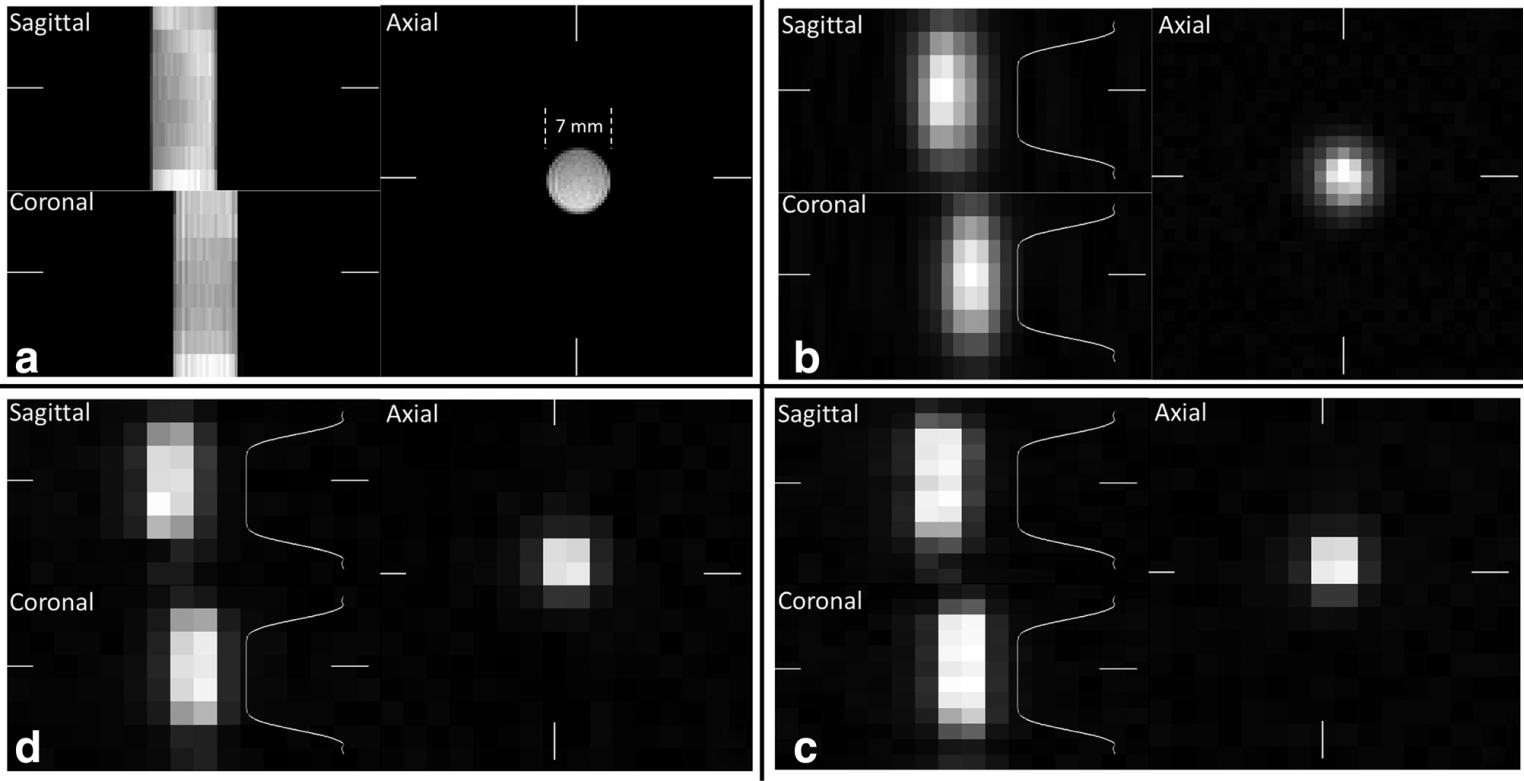
^1^H and ^13^C images (FOV of 40 × 40 × 20 mm^3^) acquired from a cylindrical phantom (inner diameter 7 mm) containing 8.5 M [1‐^13^C]lactate. Images were also acquired with a FOV in the z direction of 16 mm, 24 mm, 32 mm, and 40 mm (not shown). Proton T_2_ weighted images (**a**). ^13^C images acquired using the 32 × 32 × 8 sequence (**b**), 16 × 16 × 8 sequence (**c**), and 16 × 16 × 12 sequence (**d**). The white bars at the periphery of the images relate the position of the sagittal and coronal slices to the displayed axial slice. The white curves in the sagittal and coronal views of the ^13^C images indicate the excitation profile and its location.

Proton images, and ^13^C images acquired using the pulse sequence designs described in the methods section, of a [1‐^13^C]lactate‐containing phantom, are shown in Figure 3. The ^13^C images appear less homogeneous due to the surface receive coil. Because the ^13^C excitation slice thickness was only 13 mm, smaller than the 20 mm FOV in the z‐direction, the signal drops off toward the ends of the phantom.

**Figure 4:**
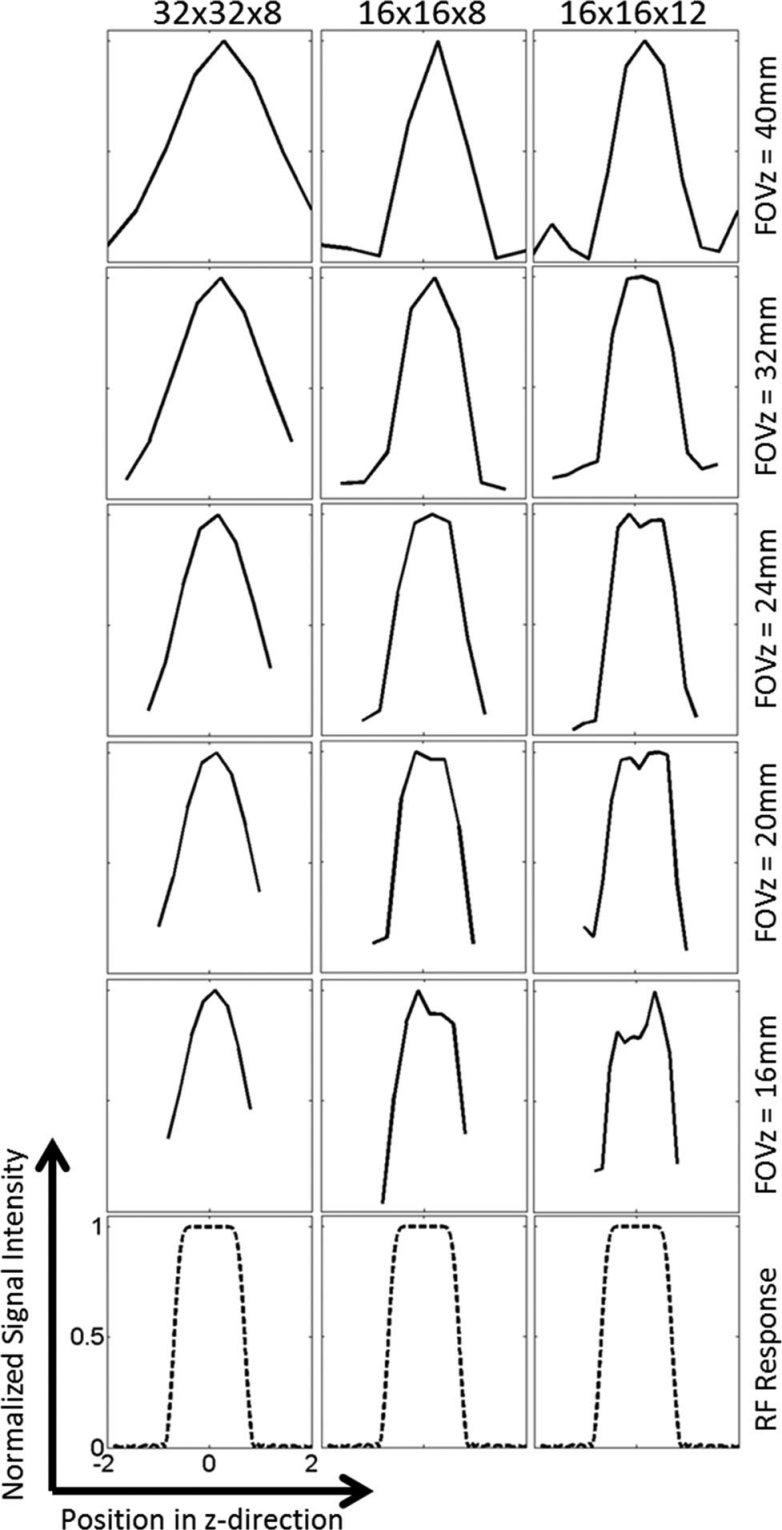
Slab profiles for a series of FOVs in the z‐direction for the three k‐space trajectory designs, compared with the simulated excitation pulse response. The slab profiles were obtained by summing over the x–y plane for images at each z position for each 3D dataset. For each subplot, the horizontal axis represents position in the z‐direction, in centimeters, and the y axis represents the signal intensity normalized to the maximum value.

**Figure 5:**
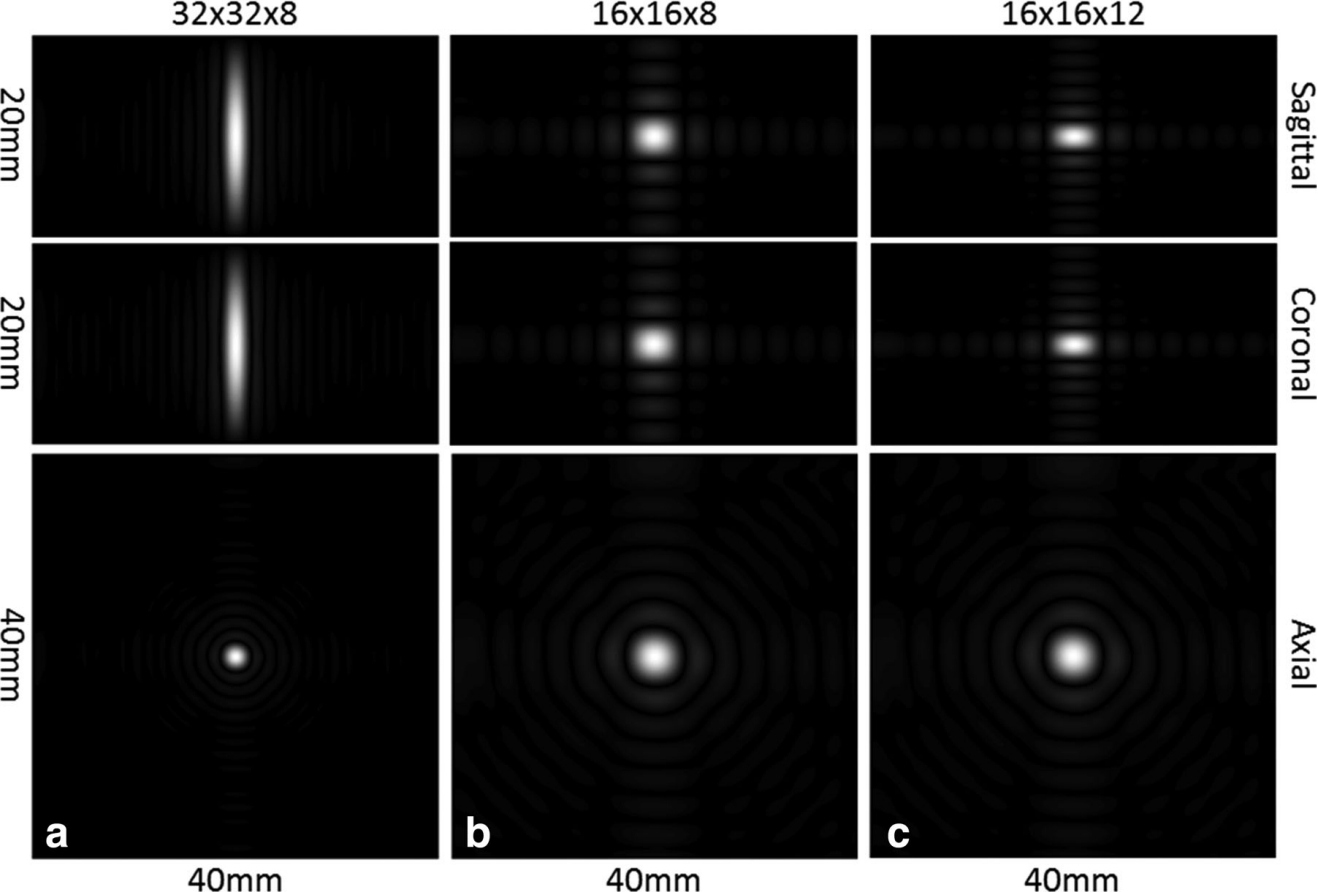
The 3D point spread functions for sagittal, coronal and axial views for the 32 × 32 × 8 (**a**), 16 × 16 × 8 (**b**) and 16 × 16 × 12 (**c**) designs. The PSF of the 16 × 16 × 12 design shows a sharp improvement in the z‐direction when compared with the 32 × 32 × 8 design, at the expense of compromised x–y plane resolution.

The slab profiles for a series of FOVs in the z‐direction, for all three spiral trajectory designs, are presented in Figure 4. These represent the combined effects of the excitation pulse, receive coil sensitivity profile, and the encoding schemes in both the x–y plane and z direction. While both the 16 × 16 × 8 and 16 × 16 × 12 designs more closely resemble the excitation profile than the 32 × 32 × 8 design, the difference is small and is probably due to the more spherical shape of the 3D k‐space in the 32 × 32 × 8 design. All three acquisition schemes produce an acceptably accurate image of the excited signal in the z‐direction. The theoretical point spread functions are shown in Figure 5.

**Figure 6:**
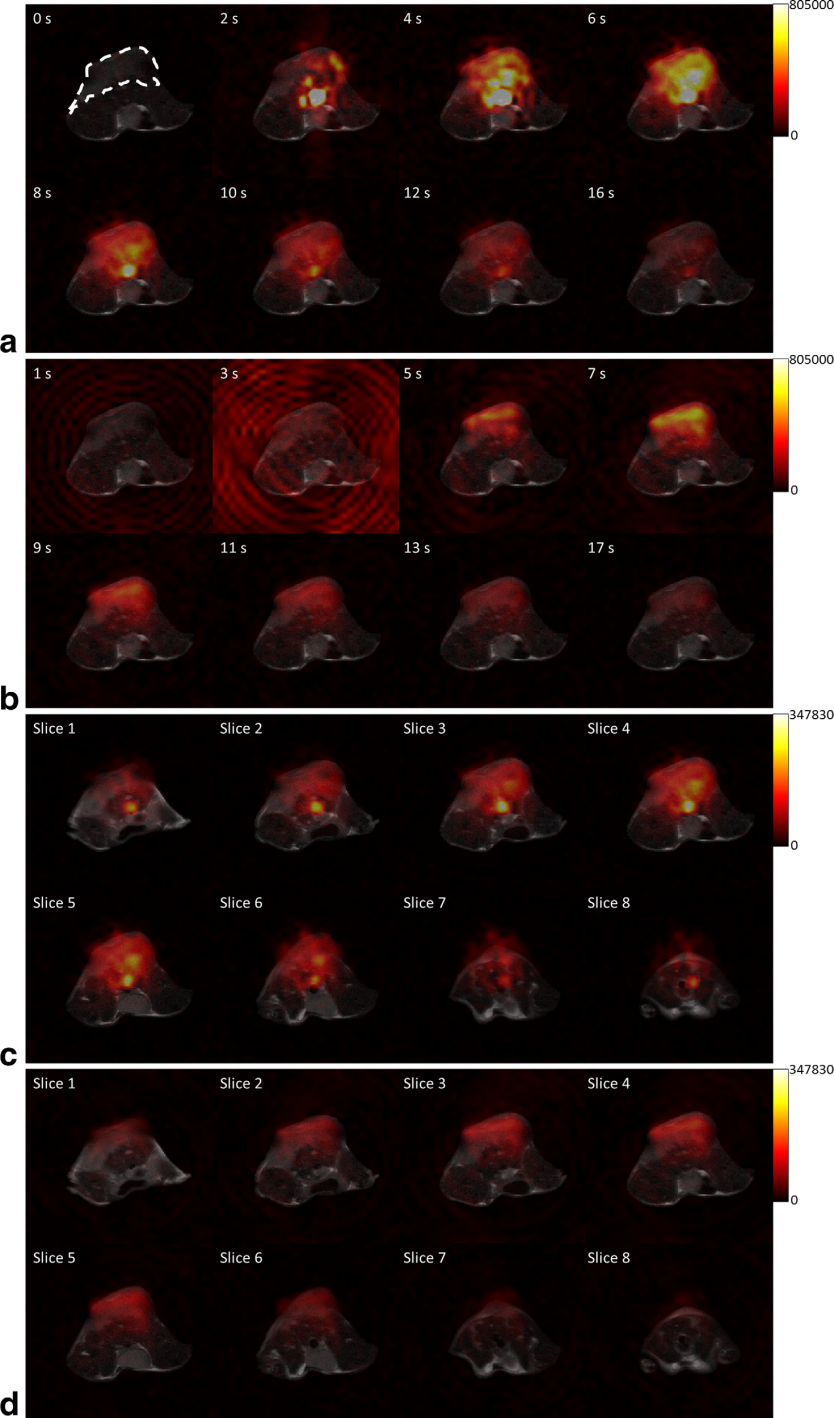
Representative dynamic hyperpolarized [1‐^13^C]pyruvate (**a**) and [1‐^13^C]lactate images (**b**) acquired using the 32 × 32 × 8 design from a single slice (4^th^), overlaid on the corresponding T_2_ weighted ^1^H image. The ^13^C images were interpolated to give a 256 × 256 in‐plane matrix, which was then overlaid on the ^1^H image, which had the same matrix size. The tumor region is outlined in the pyruvate image acquired at the first time point (a). Images from all eight slices at 6 s and 7 s are shown for pyruvate (**c**) and lactate (**d**), respectively. The images at 14 s (pyruvate) and 15 s (lactate) were acquired with the encoding gradients on the z axis turned off and were used as a reference and are not displayed. The signal intensity at each pixel is indicated with a separate color scale for each panel, with the maxima indicated in arbitrary units.

Dynamic hyperpolarized ^13^C images were acquired from three tumor‐bearing mice using the 32 × 32 × 8 design. Figure 6a shows representative [1‐^13^C]pyruvate images of the 4^th^ slice overlaid on a T_2_‐weighted ^1^H image; Figure 6b shows the corresponding [1‐^13^C]lactate image. The [1‐^13^C]pyruvate and [1‐^13^C]lactate images from all the slices at 6 s and 7 s are shown in Figures 6c and 6d, respectively. The artifacts in the lactate images at 1 and 3 s were due to some excitation of the very intense pyruvate signal in the aorta of this animal (Fig. 6b). These artifacts were not observed in the images from the other two mice.

**Figure 7:**
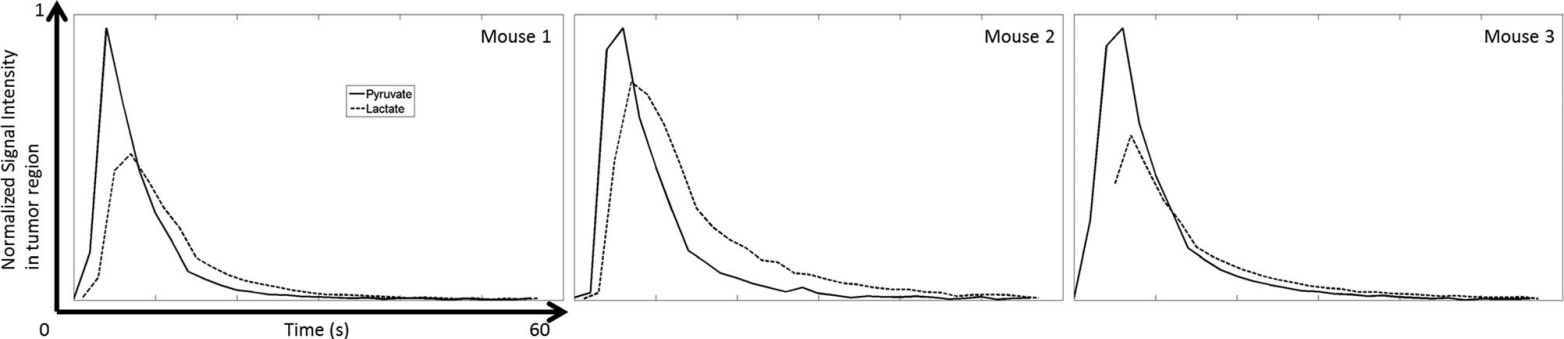
Time course of the hyperpolarized ^13^C signals from pyruvate and lactate in the tumors of the three EL4 tumor‐bearing mice. The signals were summed across all the slices, for both pyruvate (solid line) and lactate (dashed line).

**Figure 8:**
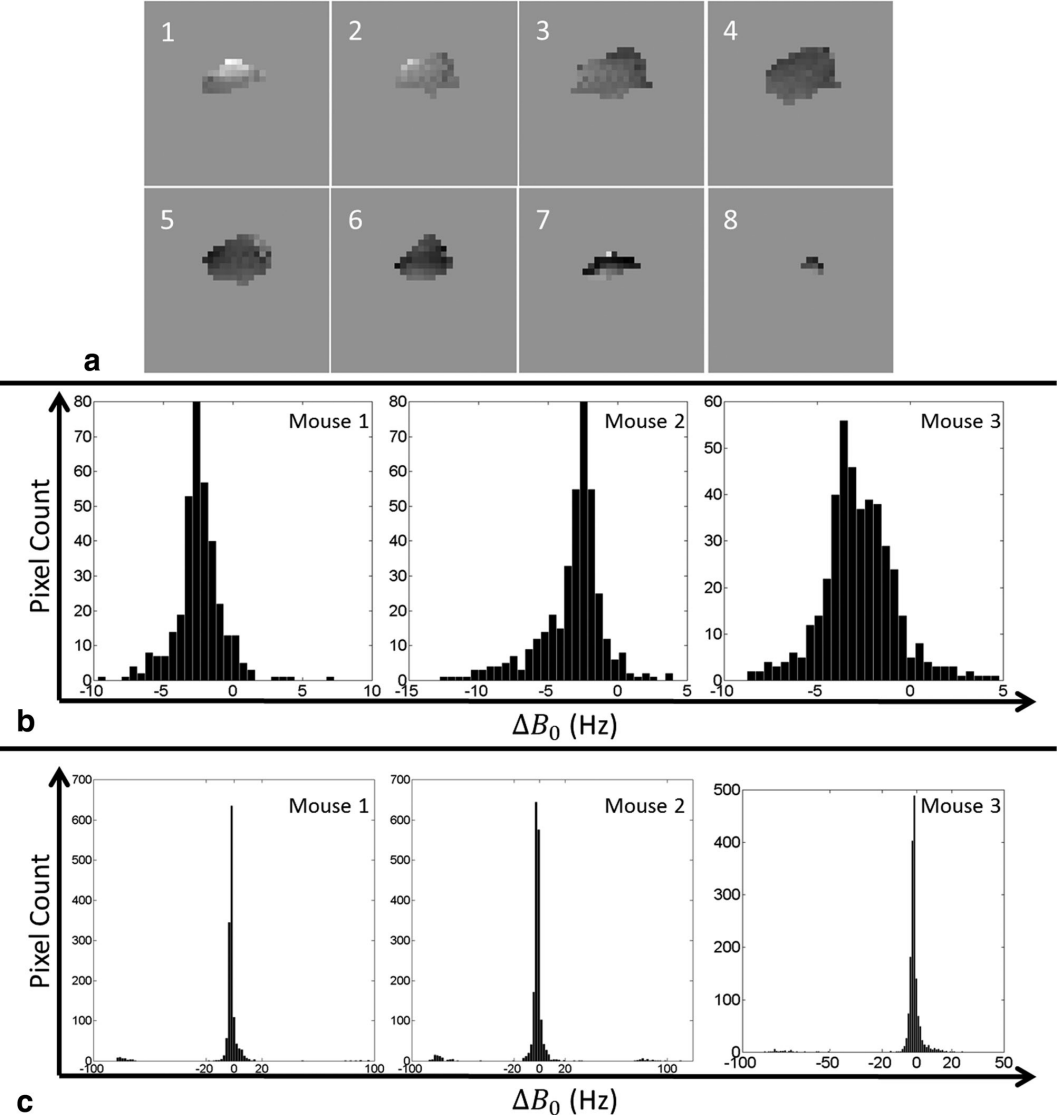
Analysis of B_0_ homogeneity. **a**: Representative B_0_ maps, expressed as ^13^C frequency variations, of the tumor region in a single mouse at all eight slice positions. The frequencies varied from ‐10 Hz to + 5 Hz, which is well below the limit of ±77.16 Hz (see the Discussion section). **b**: Histogram analysis of the B_0_ maps in the tumor regions from all three mice and for all eight slice positions. **c**: Histogram analysis of the B_0_ maps for regions of the whole animal covered by the 3D FOV. The B_0_ maps were acquired using the water proton resonance and then converted to ^13^C frequency variations based on the ^1^H and ^13^C gyromagnetic ratios.

**Figure 9:**
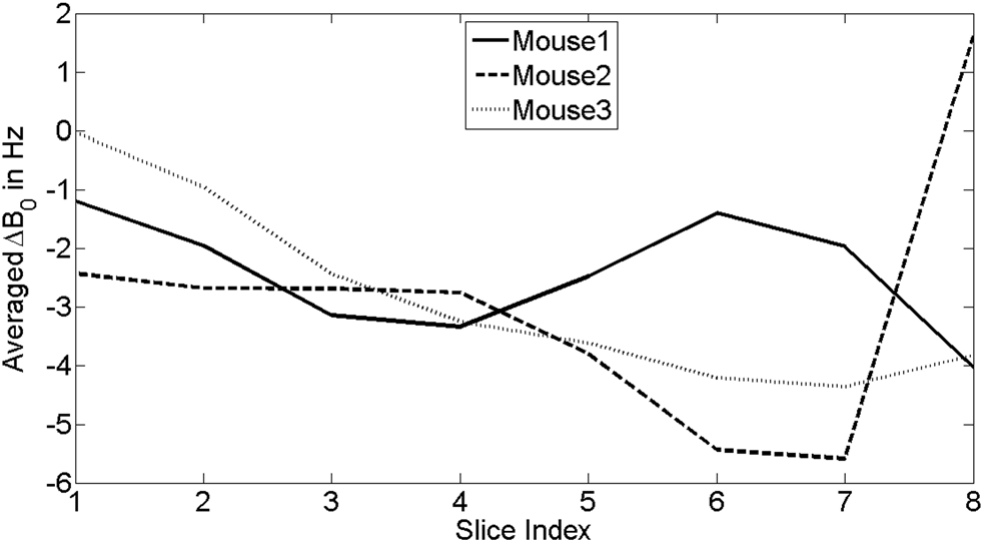
Averaged $\Delta B_{0}$ variation in the z‐direction, for all three mice. The $\Delta B_{0}$ values were averaged within the tumor region for each slice. These curves show a smooth transition of $\Delta B_{0}$ between adjacent slices and thus minimal signal modulation in the z‐direction.

The signal time courses for all three mice are shown in Figure 7. These were calculated by summing the signals from all the pixels in all the slices at each time point. The profile for the lactate signal was different from those widely reported in the literature because the 90 ° flip angle pulse on the [1‐^13^C]lactate resonance prevented accumulation of hyperpolarized ^13^C signal. The first two time points were discarded from the lactate time course for the 3^rd^ mouse because of contamination with signal from [1‐^13^C]pyruvate and [1‐^13^C]pyruvate‐hydrate (see Figure 6b). The quality of the three dimensional ^13^C images is dependent on B_0_ homogeneity over the tumor region. B_0_ maps, expressed as ^13^C frequency variations (in Hz), are shown in Figures 8a and 8b. Frequency variations in the 3D FOV covering the whole animal are shown in Figure 8c. These frequency variations are relatively small and consistent with the quality of the images shown in Figure 6. The change in ΔB_0_ with z position, averaged over each spiral, for all three mice, is shown in Figure 9.

## DISCUSSION

The pulse sequence is capable of capturing 3D information in hyperpolarized MRI experiments, after a single excitation. Compared with multishot 3D imaging techniques for hyperpolarized MRI, it minimizes the number of excitation pulses, which helps to preserve polarization, and gives a 3D acquisition in less than 125 ms, which is faster than two previously published 3D sequences that used similarly high gradient strengths [780 ms/3D image in Miller et al 21, 2.78 s/3D image in Josan et al 31]. With the sequence described here, the pyruvate and lactate polarizations can be sampled every second, which is beyond the capability of these other sequences. The sequence described here also provides comparable spatial resolution.

While the nominal spatial resolutions in 21 and 31 were 2 × 2 × 3.8 mm^3^ (matrix size 32 × 32 × 12) and 5 × 5 × 5 mm^3^ (16 × 16 × 12), the 32 × 32 × 8 design used here achieved a resolution of 1.25 × 1.25 × 2.5 mm^3^, with the 16 × 16 × 12 design achieving 2.5 × 2.5 × 1.6 mm^3^. When compared with multislice 2D imaging methods, this 3D sequence benefits from fewer excitation pulses. Although excitation pulses in 2D imaging sequences selectively excite specific slices, imperfect excitation profiles can lead to slice‐cross‐talk which further reduces the polarization in adjacent slices. Given the same flip angles, this single shot 3D imaging sequence also provides an SNR benefit over multislice 2D methods by a factor of $\sqrt{N}$ (where N is the number of phase encode steps in the z‐direction) 32, although signal decay due to T_2_ / $T_{2}^{*}$ will compromise this benefit to some extent.

This is unlike multishot 3D techniques 21, where the main SNR limitation comes from the flip angles used for each excitation. The SNR of this single shot 3D sequence could potentially be further enhanced when used with multiple averages, reduced flip angles and short TRs. The SNR enhancement in the latter case will be approximately $\sqrt{N}\times \sqrt{M}\times \frac{\sin\partial }{\sin\theta }$, where ∂ is the flip angle for each average in the 3D experiment, θ is the flip angle for the 2D experiment, and M is the number of averages. If ${\left(\cos\partial \right)}^{M}\approx \cos\theta$ then depletion of the polarization will be similar in the two experiments. A single‐shot 3D technique has been proposed previously that exploits a hybrid acquisition scheme using both fast spin echoes and an echo planar readout 33. However, when compared with the technique described here, it can have a prolonged acquisition train, due to the less efficient echo‐planar readout, and a hybrid phase error, where the stimulated echoes are mixed with the echo planar readout train.

### B_0_ inhomogeneity

Spiral readouts can suffer from off‐resonance effects. Because different z‐direction phase encoding information is involved in different spiral planes, there is no straightforward way to perform a self‐navigated spiral phase correction. The reference scan data with z‐direction phase encoding turned off was used here to correct for phase variations between spirals, and may also help to correct for off‐resonance effects within each spiral. However, all the spirals used here were very short and, therefore, accumulate only a small phase error. These ultrashort spiral readouts make off‐resonance effects less of a problem in the resulting images.

The second potential image quality problem resulting from B_0_ inhomogeneity is unique to the sequence described here. Traverses between kz steps are separated by spirals, which gives a low sampling bandwidth in the kz‐direction and can make the sequence prone to image distortions in the z‐direction. Moreover, because the spirals are of different durations, the phase errors accumulated between kz steps are different, resulting in varying distortion effects in the final image. The image distortion problem is relieved in two ways. The kz acquisition is split into two interleaved groups, which doubles the bandwidth in the kz‐direction and thus halves the potential image distortions. Second, the central two spiral planes are acquired at the spin echoes, which eliminates the phase error at the center of k‐space in the kz‐direction. At the same time, the variable duration design for the spirals in the 32 × 32 × 8 sequence keeps approximately the same image distortion, while maximizing image resolution in the x–y plane. Nevertheless, B_0_ inhomogeneity is still a potential concern but can be analyzed quantitatively as follows.

Assume that the average phase error accumulated during a spiral due to B_0_ inhomogeneity is θ(x,y,z), where x, y, and z specify spatial positions (in cm) with a certain B_0_ shift. We need to ensure that image distortion lies within a single pixel such that image splitting is not observable. For this sequence (see Appendix), the presence of B_0_ inhomogeneity ( θ(x,y,z)≠0) causes a division in the z‐direction into two images distorted by

(1) $$\pm \frac{FOVz}{2\pi }*\frac{\theta \left(x,y,z\right)}{2}$$

where FOVz is the FOV in the z direction. If this difference in position is less than a single pixel then splitting will not be observed. There is, therefore, a requirement that

(2) $$|\frac{FOVz}{2\pi }*\frac{\theta }{2}|<\frac{FOVz}{L}$$

where the right hand side is the pixel size in the z‐direction (L is the total number of encodings in the z‐direction, here 8 or 12 spirals). This requirement translates to $|\frac{L}{2\pi }*\frac{\theta }{2}|<1$, or, $|\theta |<\frac{4\pi }{L}$; when L=8, as was used in the mouse images, this becomes

(3) $$|\theta |<\frac{\pi }{2}$$

As the largest spirals in the kz‐direction, the 4^th^ and 5^th^, are aquired close to the spin echoes, and because the spiral durations vary, then it is reasonable to assume that the phase errors accumulated during the 3^rd^ or the 6^th^ spiral, both of which are 3.24 ms in duration, will be the most likely source of this z‐dimension artifact. This then puts a limit on the acceptable B_0_ shift in each single voxel

(4) $$|\Delta B_{0}\left(x,y,z\right)|<\frac{\frac{\pi }{2}}{3.24\;ms}=77.16\;Hz.$$

To check whether this condition was satisfied, spoiled gradient echo images were acquired with varying TEs and B_0_ maps calculated. The B_0_ maps of the tumor region of one mouse (Fig. 8a) showed that the B_0_ field was generally homogeneous and the variation was well under 77 Hz. This was the case for all three mice, as illustrated by the histograms of the B_0_ field distributions (Fig. 8b). In clinical systems, with more moderate gradient strengths, the proposed sequence would need to be applied together with techniques such as a restricted excitation volumes to allow a better shim.

While image distortion and ghosting are determined by the absolute value of $B_{0}$, the signal will also be modulated by the gradient of $B_{0}$ inhomogeneity in the z‐direction. Figure 9 shows the through‐slice changes of $\Delta B_{0}$ of the tumor regions, where $\Delta B_{0}$ was averaged within each slice. The overall through slice change of $\Delta B_{0}$ was less than 5 Hz, which the equations given in the appendix show would give a per pixel signal modulation of less than 5%.

### PSF

For a single shot 3D sequence, the PSF in the z‐direction determines the slice profile of the final image. A finite number of phase encoding steps in the z‐direction leads to signal leakage across the slices, and this phenomenon is exaggerated when the number of phase encoding steps is small 29. In the sequence described here, the PSF is determined not only by the number of phase encoding steps but also by the spherical shape of 3D k‐space. The gridding process used in image reconstruction can also alter the PSF. Overall, the 3D PSF resulting from the acquisition and reconstruction schemes used in the 32 × 32 × 8 implementation is shown in Figure 5a, for the sagittal, coronal, and axial views. The FWHM of the PSF in the z‐direction was approximately 7 mm, suggesting that for each slice there was significant signal contribution from other slices. In the x–y plane, the PSF has a full width at half maximum height (FWHM) of approximately 1.8 mm, which is similar to other sequences in the literature 28. Despite the compromised slice profile, the spherical‐shaped 3D k‐space has been widely used in the literature because of its high imaging efficiency 34, 35, 36, 37, 38, 39, especially in cases where ultrafast imaging is required.

The PSF in the z‐direction could be improved by trading‐off resolution in the x–y plane. Figures 5b and 5c show the PSF of the cylindrical stack of spiral trajectory designs, with a matrix size of 16 × 16 in all kx–ky planes and 8 or 12 phase encoding steps in the kz‐direction. The FWHMs of the PSFs are sharply truncated to approximately 3 mm and 2 mm in the z‐direction for the 16 × 16 × 8 and 16 × 16 × 12 designs, respectively, while in the x–y plane the FWHMs of the PSFs are more than 3 mm. The blurring of the signal in the z‐direction for each acquisition scheme can be seen in the z‐axis profiles shown in Figure 4. Each profile is principally the result of convolution of the experimental PSF, which depends on the ordering of the k‐space acquisitions in the kz‐direction, T_2_ and $T_{2}^{*}$ decays and B_0_ inhomogeneity, with the RF excitation profile. Ideally, an acquisition scheme will result in a z‐axis profile that mimics the RF excitation profile. However, in practice all these factors will affect the experimental PSF along the z‐axis leading to a stretched z‐axis profile. In the sequence implemented here Figure 3 shows a comparable z‐axis resolution for all three acquisition schemes, with minimal broadening resulting from their different theoretical PSFs. We suggest, therefore, that the acquisition scheme selected here for mouse imaging provides a practical compromise for acquiring dynamic 3D images from subcutaneous tumor models in mice.

### Limitations of the Excitation Pulse

The water proton resonance frequency was used to set the central frequencies of the ^13^C SpSp pulses. The wide spectral bandwidth (350 Hz) and the response profile of these excitation pulses enabled robust excitation of [1‐^13^C]lactate and [1‐^13^C]pyruvate. For example, a 50 Hz error in the central frequency of the [1‐^13^C]pyruvate excitation pulse will lead to only a 0.1% signal loss. However, if excitation pulses with smaller bandwidths were used, then a real time frequency measurement may be needed to achieve the desired flip angle.

A problem of using SpSp pulses to image different metabolites alternately is cross contamination of signal between the metabolites. This phenomenon is more obvious when a spiral trajectory is used for signal acquisition, as can be seen from the lactate images acquired at 1 and 3 s (Fig. 6b). The artifact in these two images, which has been observed previously 20, is the result of the SpSp pulse at the lactate resonance frequency also causing some excitation of the much more intense resonance from pyruvate (and probably from pyruvate‐hydrate as well) in the aorta. This is despite the fact that excitation of the [1‐^13^C]pyruvate and [1‐^13^C]pyruvate‐hydrate resonances is only 1% of that of the [1‐^13^C]lactate resonance. The [1‐^13^C]pyruvate signal in the aorta in this particular mouse was between 3 and 10 times more intense than the pyruvate signal in the other two mice, and hence this artifact was not observed in these other mice. The artifact was only observed in the early images, when the pyruvate signal in the aorta was much more intense than the lactate signal. However, it could be avoided altogether by using a coronal imaging slice or an excitation pulse with restricted FOV. The SpSp pulse could also be modified to further reduce cross‐talk between the pyruvate and lactate resonances, however, this could come at the expense of wider transition bands and the pulse would be less robust to the effects of B_0_ offset.

### Effects of Relaxation

For the three sequence designs, the second echo is delayed by 46–59 ms compared with the first echo. In the [1‐^13^C]lactate phantom this resulted in an approximately 5% signal decrease in the second echo. In the images acquired in vivo, the intensity of the second echo for pyruvate was 28.1 ± 8.7% lower than that of the first echo; for lactate the decrease was 12.1 ± 5.9%. These decreases in echo intensity are in agreement with the reported T_2_ values for ^13^C‐labelled pyruvate and lactate in vivo 40. If necessary, this signal loss in the second echo can be corrected for in the image reconstruction or signal analysis stages, because the echo intensities can be measured from the reference scan. As with other multiecho sequences, T_2_ and $T_{2}^{*}$‐dependent decay of the metabolite resonances broaden the PSF 41 by modulating the acquired signal in the kz direction, which is also determined by the ordering of the k‐space acquisitions. This resulted in the signal leakage observed in Figures 6c and 6d, where signal from a central slice was observed in a peripheral slice, which was evident from the presence of signal outside of the body of the animal in the peripheral slices. Although $T_{2}^{*}$ for each metabolite resonance could be estimated from the reference image this may be smaller than the true $T_{2}^{*}$ because of the effects of noise, eddy currents, and gradient/RF delays and, therefore, we did not include relaxation in calculation of the theoretical PSFs. The sequence will give higher slice resolution when used to image metabolites with longer T_2_ and $T_{2}^{*}$.

### Translation to Clinical Imaging

The sequence was designed for preclinical imaging. However, it may also find clinical applications provided that the spirals can be kept short enough to avoid image distortion in the z‐direction. With the much lower gradient strengths and slew rates used on clinical systems, this may require excitation pulses with a restricted FOV in the xy‐plane so that the desired FOV can still be fully covered by short‐duration spirals. This could be used, for example, to monitor tumor responses to treatment, where the location(s) of the tumors are already known and the excitation pulses could be focused at these sites. Spiral duration could also be kept short by extending the technique into a multishot sequence. This would still require far fewer excitations when compared with published multishot methods.

## CONCLUSIONS

We have developed a single‐shot 3D pulse sequence, based on spatial spectral pulse excitation and a stack‐of‐spirals acquisition in a multispin echo train, for hyperpolarized ^13^C MRI. The sequence was capable of imaging exchange of hyperpolarized ^13^C label between injected hyperpolarized [1‐^13^C]pyruvate and endogenous lactate in a subcutaneous tumor model with a temporal resolution of up to 250 ms and a spatial resolution of up to 1.25 mm in the x–y plane and 1.7 mm in the z‐direction. The sequence was robust to moderate B_0_ field inhomogeneity. The use of a single excitation pulse allowed for a more efficient use of the hyperpolarized magnetization. Because the sequence length was much shorter than the TR, there is the possibility for further optimization. Although the sequence was developed for preclinical imaging, it nevertheless has the potential to be used on clinical systems when the parameters are optimized for the lower gradient capabilities of these systems.

## Appendix

An analysis of the sequence's tolerance to B_0_ inhomogeneity. We define:

$$\;\{\begin{array}{c} E_{x}\;the\;phase\;accumulated\;by\;encoding\;in\;x\;direction\\ E_{y}\;the\;phase\;accumulated\;by\;encoding\;in\;y\;direction\\ E_{z}\;the\;phase\;accumulated\;by\;encoding\;in\;z\;direction \end{array}$$

where

(A1) $$E_{z}=e^{-i*2\pi *\frac{l}{FOVz}*z}$$

and FOVz is the FOV in the z‐direction. l denotes the phase encoding index in this dimension.

(A2) $$l=-\frac{L-1}{2},\;-\frac{L-3}{2},\ldots ,\;\frac{L-1}{2}.$$

The division by 2 in Eq. (A2) comes from the fact that the kx–ky plane at kz = 0 is never acquired. Instead, the kx–ky planes at kz = −0.5Δkz and 0.5Δkz are covered by the central two longest spirals.

For ρ(x,y,z), which represents the ideal image, the acquired signal in this sequence (without phase error) can be written as

(A3) $$\begin{array}{l} S\left(m,n,l\right)=\\ \;0.5*\left[∭\left\{\rho \left(x,y,z\right)*E_{x}*E_{y}*E_{z}\right\}dxdydz\right]*[1+e^{-i*\pi *(l-0.5)}]\\ \;+0.5*\left[∭\left\{\rho \left(x,y,z\right)*E_{x}*E_{y}*E_{z}\right\}dxdydz\right]*[1-e^{-i*\pi *(l-0.5)}] \end{array}$$

where m and n denote the encoding index in the x  and y directions, respectively. The two terms to sum in the equation above come from the two interleaved groups of spirals. In an acquisition with B_0_ inhomogeneity, the acquired signal becomes

(A4) $$\begin{array}{l} S^{\prime }\left(m,n,l\right)=\\ \;0.5*\left[∭\left\{\rho \left(x,y,z\right)*e^{-i*\frac{\theta }{2}*(-l+0.5)}*E_{x}*E_{y}*E_{z}\right\}dxdydz\right]*[1+e^{-i*\pi *(l-0.5)}]\\ \;+0.5*\left[∭\left\{\rho \left(x,y,z\right)*e^{-i*\frac{\theta }{2}*\left(l+0.5\right)}*E_{x}*E_{y}*E_{z}\right\}dxdydz\right]*[1-e^{-i*\pi *(l-0.5)}] \end{array}$$

The average phase error θ(x,y,z) is divided by 2 because of the interleaved kz‐dimension acquisition. In Eq. (A4), the phase error accumulation for the first group of spirals ( l=−3.5, −1.5, 0.5, 2.5) is maximum for the 1^st^ spiral, less for the 2^nd^ spiral, fully refocused at the 3^rd^ spiral (where the spin echo is formed), and greater again for the 4^th^ spiral. The same trend is maintained for the second group of spirals ( l=3.5, 1.5, −0.5, −2.5). Eq. (A4) can be rewritten as the summation of four terms

(A5) $$\begin{array}{l} S^{\prime }\left(m,n,l\right)=\\ \;0.5*\left[∭\left\{\rho \left(x,y,z\right)*E_{x}*E_{y}*e^{-i*2\pi *\frac{l}{FOVz}*\left(z-\frac{\theta /2}{2\pi }*FOVz\right)}*e^{-i*\frac{\theta }{4}}\right\}dxdydz\right]\\ \;+0.5*\left[∭\left\{\rho \left(x,y,z\right)*E_{x}*E_{y}*e^{-i*2\pi *\frac{l}{FOVz}*\left(z-\frac{\theta /2}{2\pi }*FOVz+\frac{FOVz}{2}\right)}*e^{-i*\frac{\theta }{4}}*e^{i*\frac{\pi }{2}}\right\}dxdydz\right]\\ \;+0.5*\left[∭\left\{\rho \left(x,y,z\right)*E_{x}*E_{y}*e^{-i*2\pi *\frac{l}{FOVz}*\left(z+\frac{\theta /2}{2\pi }*FOVz\right)}*e^{-i*\frac{\theta }{4}}\right\}dxdydz\right]\\ \;-0.5*\left[∭\left\{\rho \left(x,y,z\right)*E_{x}*E_{y}*e^{-i*2\pi *\frac{l}{FOVz}*\left(z+\frac{\theta /2}{2\pi }*FOVz+\frac{FOVz}{2}\right)}*e^{-i*\frac{\theta }{4}}*e^{i*\frac{\pi }{2}}\right\}dxdydz\right] \end{array}$$

Each term in Eq. (A5) can be reconstructed into a separate image modulated from the ideal image, similar to the analysis in Xu et al 42. For the first term of the four terms in Eq. (A5), let

(A6) $$z^{\prime }=z-\frac{\theta /2}{2\pi }*FOVz=\beta (z)$$

we then have

(A7) $$z=\beta^{-1}(z^{\prime })$$

Let $S^{\prime }{}_{1}(m,n,l)$ represent the first term in Eq. (A5). We can now rewrite this first term as a function of z′

(A8) $$\begin{array}{l} S^{\prime }{}_{1}\left(m,n,l\right)\\ \;=∭\left\{\rho \left[x,y,\beta^{-1}(z^{\prime })\right]*E_{x}*E_{y}*e^{-i*2\pi *\frac{l}{FOVz}*z^{\prime }}*e^{-i*\frac{\theta }{4}}*\frac{\partial \beta^{-1}(z^{\prime })}{\partial z^{\prime }}\right\}dz^{\prime }dydx \end{array}$$

A 3D Fourier transform of $S^{\prime }{}_{1}(m,n,l)$ yields the first separate reconstructed image

(A9) $$\rho^{\prime }{}_{1}\left(x,y,z^{\prime }\right)=\rho \left[x,y,\beta^{-1}(z^{\prime })\right]*\frac{\partial \beta^{-1}\left(z^{\prime }\right)}{\partial z^{\prime }}*e^{-i*\frac{\theta }{4}}$$

By substituting variable z (Eqs. (A6) and (A7)) back into Eq. (A9), we get

(A10) $$\begin{array}{l} \;\rho^{\prime }{}_{1}\left(x,y,z-\frac{FOVz}{2\pi }*\frac{\theta }{2}\right)\\ \;=\rho \left(x,y,z\right)/\left[1-\frac{FOVz}{2\pi }*\frac{\partial \theta \left(x,y,z\right)/2}{\partial z}\right]*e^{-i*\frac{\theta }{4}} \end{array}$$

Similar operations can be performed on the rest of the three terms in Eq. (A5), giving Eq. (A11).

(A11) $$\{\begin{array}{l} \rho^{\prime }{}_{1}\left(x,y,z-\frac{FOVz}{2\pi }*\frac{\theta }{2}\right)=\rho \left(x,y,z\right)/\left[1-\frac{FOVz}{2\pi }*\frac{\partial \theta \left(x,y,z\right)/2}{\partial z}\right]*e^{-i*\frac{\theta }{4}}\\ \rho^{\prime }{}_{2}\left(x,y,z-\frac{FOVz}{2\pi }*\frac{\theta }{2}+\frac{FOVz}{2}\right)=\rho \left(x,y,z\right)/\left[1-\frac{FOVz}{2\pi }*\frac{\partial \theta \left(x,y,z\right)/2}{\partial z}\right]*e^{-i*\frac{\theta }{4}}*e^{i*\frac{\pi }{2}}\\ \rho^{\prime }{}_{3}\left(x,y,z+\frac{FOVz}{2\pi }*\frac{\theta }{2}\right)=\rho \left(x,y,z\right)/\left[1+\frac{FOVz}{2\pi }*\frac{\partial \theta \left(x,y,z\right)/2}{\partial z}\right]*e^{-i*\frac{\theta }{4}}\\ \rho^{\prime }{}_{4}\left(x,y,z+\frac{FOVz}{2\pi }*\frac{\theta }{2}+\frac{FOVz}{2}\right)=-\rho \left(x,y,z\right)/\left[1+\frac{FOVz}{2\pi }*\frac{\partial \theta \left(x,y,z\right)/2}{\partial z}\right]*e^{-i*\frac{\theta }{4}}*e^{i*\frac{\pi }{2}} \end{array}$$

The overall reconstructed image (or, observed image) ρ′(x,y,z) is the summation of the four separately reconstructed images:

(A12) $$\;\rho^{\prime }\left(x,y,z\right)=0.5*[\rho_{1}^{\prime }\left(x,y,z\right)+\rho_{2}^{\prime }\left(x,y,z\right)+\rho_{3}^{\prime }\left(x,y,z\right)+\rho_{4}^{\prime }\left(x,y,z\right)]$$

In an ideal case, where the B_0_ field is absolutely homogeneous ( θ(x,y,z)=0 for any x, y, and z), the observed image is identical to the ideal image (or, $\rho^{\prime }\left(x,y,z\right)=\rho (x,y,z)$). This can be proved by substituting Eq. (A11) into Eq. (A12) with θ=0. In the presence of B_0_ inhomogeneity ( θ(x,y,z)≠0 and θ varies with x, y, and z), the observed image is split (in the z‐direction) into two images, distorted by the term given in Eq. (1) in the main text and each has a ghost at the $\frac{FOVz}{2}$ position in the z direction. Note that the ghostings from the split images have opposite signs. As long as the condition in Eq. (1) in the main text is met, the image distortion is within a single pixel such that the image splitting is unobservable and the ghosts from the split images will cancel each other.

## References

[1] Ardenkjaer JH , Fridlund B , Gram A , Hansson G , Hannson L , Lerche MH , Servin R , Thaning M , Golman K . Increase in signal‐to‐noise ratio of > 10,000 times in liquid state NMR. Proc Natl Acad Sci U S A 2003;100:10158–10163. 12930897 10.1073/pnas.1733835100 PMC193532

[2] Brindle KM . New approaches for imaging tumour responses to treatment. Nat Rev Cancer 2008;8:94–107. 18202697 10.1038/nrc2289

[3] Brindle KM . Imaging metabolism with hyperpolarized ^13^C‐labeled cell substrates. J Am Chem Soc 2015;137:6418–6427. 25950268 10.1021/jacs.5b03300

[4] Nelson SJ , Kurhanewicz J , Vigneron DB , et al. Metabolic imaging of patients with prostate cancer using hyperpolarized [1‐^13^C]pyruvate. Sci Transl Med 2013;5:198ra108. 10.1126/scitranslmed.3006070 PMC4201045 23946197

[5] Brindle KM , Bohndiek SE , Gallagher FA , Kettunen MI . Tumor imaging using hyperpolarized ^13^C magnetic resonance spectroscopy. Magn Reson Med 2011;66:505–519. 21661043 10.1002/mrm.22999

[6] Day SE , Kettunen MI , Gallagher FA , Hu DE , Lerche M , Wolber J , Golman K , Ardenkjaer‐Larsen JH , Brindle KM . Detecting tumor response to treatment using hyperpolarized ^13^C magnetic resonance imaging and spectroscopy. Nat Med 2007;13:1382–1387. 17965722 10.1038/nm1650

[7] Brindle KM . Watching tumours gasp and die with MRI: the promise of hyperpolarized ^13^C MR spectroscopic imaging. Br J Radiol 2012;85:697–708. 22496072 10.1259/bjr/81120511 PMC3474112

[8] Cunningham CH , Vigneron DB , Chen AP , Xu D , Nelson SJ , Hurd RE , Kelley DA , Pauly JM . Design of flyback echo‐planar readout gradients for magnetic resonance spectroscopic imaging. Magn Reson Med 2005;54:1286–1289. 16187273 10.1002/mrm.20663

[9] Cunningham CH , Chen AP , Albers MJ , Kurhanewicz J , Hurd RE , Yen YF , Pauly JM , Nelson SJ , Vigneron DB . Double spin‐echo sequence for rapid spectroscopic imaging of hyperpolarized ^13^C. J Magn Reson 2007;187:357–362. 17562376 10.1016/j.jmr.2007.05.014

[10] Larson PEZ , Bok R , Kerr AB , Lustig M , Hu S , Chen AP , Nelson SJ , Pauly JM , Kurhanewicz J , Vigneron DB . Multiband excitation pulses for hyperpolarized ^13^C dynamic chemical‐shift imaging. J Magn Reson 2008;194:121–127. 18619875 10.1016/j.jmr.2008.06.010 PMC3739981

[11] Larson PEZ , Bok R , Kerr AB , Lustig M , Hu S , Chen AP , Nelson SJ , Pauly JM , Kurhanewicz J , Vigneron DB . Investigation of tumor hyperpolarized [1‐^13^C]‐pyruvate dynamics using time‐resolved multiband RF excitation echo‐planar MRSI. Magn Reson Med 2010;63:582–591. 20187172 10.1002/mrm.22264 PMC2844437

[12] Hu S , Lustig M , Chen AP , et al. Compressed sensing for resolution enhancement of hyperpolarized ^13^C flyback 3D‐MRSI. J Magn Reson 2008;192:258–264. 18367420 10.1016/j.jmr.2008.03.003 PMC2475338

[13] Hu S , Lustig M , Balakrishnan A , Larson PE , Bok R , Kurhanewicz J , Nelson SJ , Goga A , Pauly JM , Vigneron DB . 3D compressed sensing for highly accelerated hyperpolarized ^13^C MRSI with in vivo applications to transgenic mouse models of cancer. Magn Reson Med 2010;63:312–321. 20017160 10.1002/mrm.22233 PMC2829256

[14] Larson PE , Hu S , Lustig M , Kerr AB , Nelson SJ , Kurhanewicz J , Pauly JM , Vigneron DB . Fast dynamic 3D MR spectroscopic imaging with compressed sensing and multiband excitation pulses for hyperpolarized ^13^C studies. Magn Reson Med 2011;65:610–619. 20939089 10.1002/mrm.22650 PMC3021589

[15] Levin YS , Mayer D , Yen YF , Hurd RE , Spielman DM . Optimization of fast spiral chemical shift imaging using least squares reconstruction: application for hyperpolarized ^13^C metabolic imaging. Magn Reson Med 2007;58:245–252. 17654596 10.1002/mrm.21327

[16] Mayer D , Yen YF , Tropp J , Pfefferbaum A , Hurd RE , Spielman DM . Application of subsecond spiral chemical shift imaging to real‐time multislice metabolic imaging of the rat in vivo after injection of hyperpolarized ^13^C‐pyruate. Magn Reson Med 2009;62:557–564. 19585607 10.1002/mrm.22041 PMC2782691

[17] Mayer D , Yen YF , Takahashi A , Josan S , Tropp J , Rutt BK , Hurd RE , Spielman DM , Pfefferbaum A . Dynamic and high‐resolution metabolic imaging of hyperpolarized [1‐^13^C]‐pyruvate in the rat brain using a high‐performance gradient insert. Magn Reson Med 2011;65:1228–1233. 21500253 10.1002/mrm.22707 PMC3126907

[18] Cunningham CH , Chen AP , Lustig M , et al. Pulse sequence for dynamic volumetric imaging of hyperpolarized metabolic products. J Magn Reson 2008;193:139–146. 18424203 10.1016/j.jmr.2008.03.012 PMC3051833

[19] Lau AZ , Chen AP , Ghugre NR , Ramanan V , Lam WW , Connelly KA , Wright GA , Cunningham CH . Rapid multislice imaging of hyperpolarized ^13^C pyruvate and bicarbonate in the heart. Magn Reson Med 2010;64:1323–1331. 20574989 10.1002/mrm.22525

[20] Schulte RF , Sperl JI , Weidl E , et al. Saturation‐recovery metabolic‐exchange rate imaging with hyperpolarized [1‐^13^C]pyruvate using spectral‐spatial excitation. Magn Reson Med 2012;69:1209–1216. 22648928 10.1002/mrm.24353

[21] Miller JJ , Lau AZ , Teh I , Schneider JE , Kinchesh P , Smart S , Ball V , Sibson NR , Tyler DJ . Robust and high resolution hyperpolarized metabolic imaging of the rat heart at 7 T with 3D spectral‐spatial EPI. Magn Reson Med 2016;75:1515–1524. 25991606 10.1002/mrm.25730 PMC4556070

[22] Wiesinger F , Weidl E , Menzel MI , Janich MA , Khegai O , Glaser SJ , Haase A , Schwaiger M , Schulte RF . IDEAL spiral CSI for dynamic metabolic MR imaging of hyperpolarized [1‐^13^C]pyruvate. Magn Reson Med 2012;68:8–16. 22127962 10.1002/mrm.23212

[23] Bedard PL , Hansen AR , Ratain MJ , Siu LL . Tumour heterogeneity in the clinic. Nature 2013;501:355–364. 24048068 10.1038/nature12627 PMC5224525

[24] Junttila MR , de Sauvage FJ . Influence of tumour micro‐environment heterogeneity on therapeutic response. Nature 2013;501:346–354. 24048067 10.1038/nature12626

[25] Pauly J , Nishimura D , Macovski A . A k‐space analysis of small tip excitation. J Magn Reson 1989;81:43–56. 10.1016/j.jmr.2011.09.023 22152370

[26] Pauly J , Le Roux P , Nishimura D , Macovski A . Parameter relations for the Shinnar‐Le Roux RF pulse design algorithm. IEEE Trans Med Imaging 1991;10:53–65. 18222800 10.1109/42.75611

[27] Glover G . Simple analytic spiral k‐space algorithm. Magn Reson Med 1999;42:412–415. 10440968 10.1002/(sici)1522-2594(199908)42:2<412::aid-mrm25>3.0.co;2-u

[28] Durst M , Koellisch U , Frank A , et al. Comparison of acquisition schemes for hyperpolarised ^13^C imaging. NMR Biomed 2015;28:715–725. 25908233 10.1002/nbm.3301

[29] Lai S , Glover GH . Three‐dimensional spiral fMRI technique: a comparison with 2D spiral acquisition. Magn Reson Med 1998;39:68–78. 9438439 10.1002/mrm.1910390112

[30] Beatty PJ , Nishimura DG , Pauly JM . Rapid gridding reconstruction with a minimal oversampling ratio. IEEE Trans Med Imaging 2005;24:799–808. 15959939 10.1109/TMI.2005.848376

[31] Josan S , Hurd R , Park JM , Yen YF , Watkins R , Pfefferbaum A , Spielman D , Mayer D . Dynamic metabolic imaging of hyperpolarized [2‐^13^C]pyruvate using spiral chemical shift imaging with alternating spectral band excitation. Magn Reson Med 2014;71:2051–2058. 23878057 10.1002/mrm.24871 PMC3849119

[32] Bernstein MA , King KF , Zhou XJ . Three‐dimensional acquisition In: Handbook of MRI pulse sequences. Amsterdam: Elsevier Academic Press; 2004. p 429.

[33] Sukumar S , Hu S , Larson PE , Zhang VY , Ohliger M , Bok R , Kurhanewicz J , Vigneron DB . Single‐shot, 2D and 3D dynamic imaging of hyperpolarized ^13^C biomarkers in vivo at 14.1 Tesla. In Proceedings of the 20th Annual Meeting of ISMRM, Melbourne, Australia, 2012. Abstract 4292.

[34] van Gorp JS , Bakker CJ , Bouwman JG , Smink J , Zijlstra F , Seevinck PR . Geometrically undistorted MRI in the presence of field inhomogeneities using compressed sensing accelerated broadband 3D phase encoded turbo spin‐echo imaging. Phys Med Biol 2015;60:615–631. 25548990 10.1088/0031-9155/60/2/615

[35] Wu HH , Nishimura DG . 3D Magnetization‐prepared imaging using a stack‐of‐rings trajectory. Magn Reson Med 2010;63:1210–1218. 20432292 10.1002/mrm.22288 PMC2905147

[36] Shu Y , Riederer SJ , Bernstein MA . Three‐dimensional MRI with an undersampled spherical shells trajectory. Magn Reson Med 2006;56:553–562. 16894580 10.1002/mrm.20977

[37] Irarrazabal P , Nishimura DG . Fast three dimensional magnetic resonance imaging. Magn Reson Med 1995;33:656–662. 7596269 10.1002/mrm.1910330510

[38] Irarrazabal P , Santos JM , Guarini M , Nishimura D . Flow properties of fast three‐dimensional sequences for MR angiography. Magn Reson Imaging 1999;17:1469–1479. 10609995 10.1016/s0730-725x(99)00083-1

[39] Asslander J , Zahneisen B , Hugger T , Reisert M , Lee HL , LeVan P , Hennig J . Single shot whole brain imaging using spherical stack of spirals trajectories. Neuroimage 2013;73:59–70. 23384526 10.1016/j.neuroimage.2013.01.065

[40] Kettunen MI , Kennedy BW , Hu DE , Brindle KM . Spin echo measurements of the extravasation and tumor cell uptake of hyperpolarized [1‐^13^C]lactate and [1‐^13^C]pyruvate. Magn Reson Med 2013;70:1200–1209. 23280500 10.1002/mrm.24591

[41] Qin Q . Point spread functions of the T2 decay in k‐space trajectories with long echo train. Magn Reson Imaging 2012;30:1134–1142. 22817958 10.1016/j.mri.2012.04.017 PMC3443331

[42] Xu D , Maier JK , King KF , Collick BD , Wu G , Peters RD , Hinks RS . Prospective and retrospective high order eddy current mitigation for diffusion weighted echo planar imaging. Magn Reson Med 2013;70:1293–1305. 23325564 10.1002/mrm.24589

